# Immigrant Health Inequalities in the United States: Use of Eight Major National Data Systems

**DOI:** 10.1155/2013/512313

**Published:** 2013-10-27

**Authors:** Gopal K. Singh, Alfonso Rodriguez-Lainz, Michael D. Kogan

**Affiliations:** ^1^US Department of Health and Human Services, Health Resources and Services Administration, Maternal and Child Health Bureau, 5600 Fishers Lane, Room 18-41, Rockville, MD 20857, USA; ^2^Centers for Disease Control and Prevention, Division of Global Migration and Quarantine, 3851 Rosecrans Street, Mailstop P575, Suite 715, San Diego, CA 92110, USA

## Abstract

Eight major federal data systems, including the National Vital Statistics System (NVSS), National Health Interview Survey (NHIS), National Survey of Children's Health, National Longitudinal Mortality Study, and American Community Survey, were used to examine health differentials between immigrants and the US-born across the life course. Survival and logistic regression, prevalence, and age-adjusted death rates were used to examine differentials. Although these data systems vary considerably in their coverage of health and behavioral characteristics, ethnic-immigrant groups, and time periods, they all serve as important research databases for understanding the health of US immigrants. The NVSS and NHIS, the two most important data systems, include a wide range of health variables and many racial/ethnic and immigrant groups. Immigrants live 3.4 years longer than the US-born, with a life expectancy ranging from 83.0 years for Asian/Pacific Islander immigrants to 69.2 years for US-born blacks. Overall, immigrants have better infant, child, and adult health and lower disability and mortality rates than the US-born, with immigrant health patterns varying across racial/ethnic groups. Immigrant children and adults, however, fare substantially worse than the US-born in health insurance coverage and access to preventive health services. Suggestions and new directions are offered for improvements in health monitoring and for strengthening and developing databases for immigrant health assessment in the USA.

## 1. Introduction

The US immigrant population has grown considerably in the last four decades, from 9.6 million in 1970 to 40.4 million in 2011 [1–5]. Immigrants currently represent 13.0% of the total US population, the highest percentage in eight decades [1, 5]. The rapid increase in the immigrant population since 1970 reflects large-scale immigration from Latin America and Asia [1–3]. Over half (53%) of all US immigrants are from Latin America, and another 29% of immigrants come from Asia [1, 5]. Europeans, who accounted for 75% of immigrants in 1960, currently represent 12% of the total US immigrant population [1, 5]. There are currently 29.2 million immigrants in the prime work force (ages 25–64 years), making up about 17.7% of the total US population [1, 5]. The number of US children in immigrant families more than doubled in the past two decades, from 8.2 million in 1990 to 17.5 million in 2011 [5, 6]. In 2011, nearly a quarter of US children had at least one foreign-born parent [5, 6]. 

Despite the marked increase in the population, the systematic monitoring of health, mortality, and disease patterns among US immigrant populations of various ethnic and national origins remains relatively uncommon [7, 8]. Most national data systems in the US do not routinely report and analyze health statistics by immigrant status. Moreover, immigrant health analysis is hampered by difficulty in obtaining relevant population denominator data or by an incomplete reporting of immigrant status in national surveillance databases [7, 8]. The substantial ethnic, cultural, and linguistic diversity of the US immigrant population makes it even more difficult to monitor immigrant health and well-being on a systematic basis [7, 8].

Although reduction of health inequalities among various sociodemographic groups remains the primary focus of *Healthy People*, this national health initiative in health promotion and disease prevention lacks data or policy objectives that explicitly target the health of US immigrants [9–11]. Moreover, the nation's premier and most comprehensive annual report on health statistics, *Health*, *United States*, does not include any data on the US immigrant population [12]. 

In this study, we describe eight major federal data systems that can be used to study the health of immigrants in the US in considerable detail. These data systems vary considerably in their coverage of health and behavioral characteristics, identification of major immigrant groups, and availability of time periods. A second, equally important objective is to provide, by using these data systems, contemporary estimates of some of the most important health and behavioral indicators for both immigrant and US-born populations across the life course, including life expectancy, infant mortality, low birthweight, mortality from major causes of death such as cancers, cardiovascular diseases (CVD), homicide, suicide, and unintentional injuries, self-assessed physical and mental health, disability, health insurance coverage, and health-risk factors such as smoking, obesity, and physical inactivity. We discuss the relative significance of each data system for carrying out immigrant health analyses in the US and offering suggestions and new directions for strengthening and developing databases for immigrant health assessment.

## 2. Methods

Strengths, limitations, and characteristics of each data system are summarized in Table 1. Survival and logistic regression models, prevalence, age-specific and age-adjusted death rates, and standard life table methodology are used to examine nativity/immigrant differentials. Since all health surveys discussed in this study have complex sampling designs, SUDAAN software is used to estimate prevalence, standard errors, and regression models [14]. Where possible, nativity differentials in health and disease outcomes are adjusted for relevant socioeconomic and demographic characteristics. The complete count administrative data systems are described and analyzed first, followed by the national sample surveys, broadly adopting a life course perspective.

### 2.1. National Vital Statistics System (NVSS)

The NVSS has long been the cornerstone of health monitoring among socio-demographic groups and geographic areas in the US for over a century [16–20]. The NVSS is a vital registration system of all births and deaths occurring in the US [16, 17]. The system is maintained by the Centers for Disease Control's (CDC's), National Center for Health Statistics (NCHS). The national mortality data are available on an annual basis in published form from 1900 to present and on public-use microdata files from 1968 to 2010 [16, 18]. This data system allows the examination of mortality differentials by cause of death according to individual characteristics, including nativity/immigrant status and geographic areas such as states, counties, and metropolitan/nonmetropolitan areas. The national mortality data system is one of the very few administrative sources of health statistics in the US that is routinely available, that covers all events, and that is comparable at the international, national, state, and local levels [18, 19].

The national mortality files are based on information from death certificates of every death occurring in the United States each year. In 2010, 2,468,435 deaths were reported in the US [21]. The *US Standard Certificate of Death*, revised most recently in 2003, is the basis for the national mortality data [16, 21]. 

For the study of mortality differentials, the following variables are available on the death certificate: sex, race/ethnicity, age at death, place/country-of-birth of decedent, place of residence, educational attainment, occupation, industry, and marital status of decedent, underlying and multiple causes of death (coded according to the *International Classification of Diseases*), autopsy status, place of death (hospital, clinic, nursing home, residence, etc.), and injury at work [16, 21].

Nativity/immigrant status in the mortality file is determined by decedent's state/country of birth [7, 8, 21]. The place-of-birth variable includes codes for the 50 states, the District of Columbia (DC), US territories of Puerto Rico, Virgin Islands, Guam, American Samoa, and Northern Marianas, and those born in Canada, Mexico, Cuba, and the remainder of the world [21]. For mortality analysis, those born outside the 50 states, DC, and US territories are considered foreign-born [7, 8]. In 2010, 209,512 deaths occurred among the foreign-born, representing 8.5% of all US deaths. About 13,000 deaths occurred among those born in Canada, while 33,898 deaths occurred among those born in Mexico [21]. In 2010, 0.7% of the death records had missing state/country-of-birth information. For computing mortality rates, relevant population (denominator) data on nativity/immigrant status, race/ethnicity, and sociodemographic characteristics can be obtained from the decennial censuses or the American Community Survey [7, 8, 16].

The major advantages of the national mortality file are its size, geographic and ethnic detail, and the fact that the information on individual death records is available electronically since 1968 [7, 18, 19]. Moreover, the availability of published information since 1900 on an annual basis makes it especially useful for analyzing long-term national and state trends in mortality, survival, and life expectancy [16, 18, 19].

The natality component of the NVSS includes birth certificate data for over 4 million births that occur in the United States each year [12, 17, 22]. Birth-certificate data are available on an annual basis in published form from 1915 to present and in electronic form on public-use data files from 1968 to 2010 [17, 22]. The *US Standard Certificate of Live Birth*, revised most recently in 2003, is the basis for the national birth data [17]. 

Nativity/immigrant status of infants and mothers in the natality file is defined according to the mother's place (state/country) of birth. The place-of-birth variable in the natality file is identical to that in the mortality file. However, for birth data, detailed codes for the mother's country of birth are also available [22]. Out of 4.0 million US births in 2010, 930,135 births occurred among foreign-born mothers. In 2010, 356,125 births occurred among mothers born in Mexico, 33,711 births among mothers born in India, 23,227 births among mothers born in China, 22,285 births among mothers born in the Philippines, and 10,612 births among mothers born in Canada [22]. In 2010, 0.3% of US birth records lacked place-of-birth information.

Besides nativity/immigrant status, the variables available for analyzing fertility and birth outcomes include maternal and paternal age, race/ethnicity, marital status, education, birthweight, gestational age, tobacco and alcohol use during pregnancy, prenatal care utilization, maternal weight gain during pregnancy, method of delivery (vaginal or c-section), pregnancy history, and a variety of medical risk factors and complications such as gestational diabetes, pregnancy-induced hypertension, eclampsia, uterine bleeding, and placenta previa [17, 22].

#### 2.1.1. Selected Results

**Figure 1:**
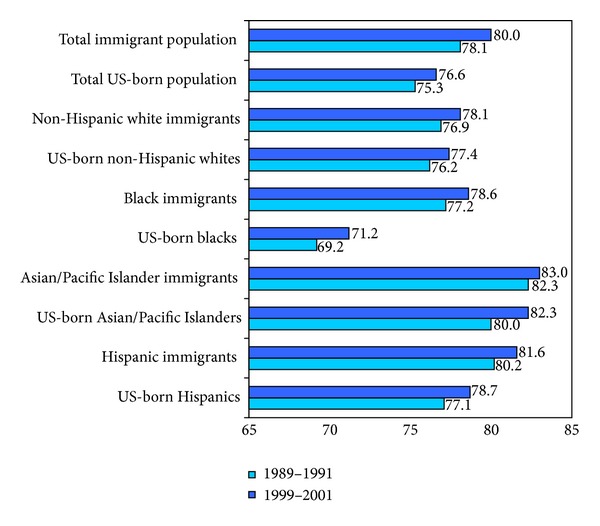
Life expectancy at birth (average lifetime in years) by race/ethnicity and immigrant status, United States, 1989–2001. Source: based on data from the US National Vital Statistics System, 1989–2001. Also, see [7].

Selected immigrant health patterns based on US mortality data are shown in Figure 1 and Table 2. During 1999–2001, US immigrants had a life expectancy of 80.0 years, 3.4 years longer than the life expectancy of the US-born population (Figure 1). Nativity differentials in life expectancy increased between 1989 and 2001. In all racial/ethnic groups, immigrants had a higher life expectancy than their US-born counterparts. The nativity differential was greatest for black immigrants who had 7.4 years longer life expectancy than US-born blacks. Among the foreign-born population, Asian/Pacific Islander (API) immigrants had the highest life expectancy (83.0 years), followed by Hispanic immigrants (81.6 years), black immigrants (78.6 years), and white immigrants (78.1 years) [7]. 

During 1999–2001, male and female immigrants experienced 23% and 16% lower all-cause mortality than their US-born counterparts, respectively (Table 2). This pattern held for whites, blacks, APIs, and Hispanics. Ethnic-nativity patterns in CVD and all-cancer mortality were generally similar to those in all-cause mortality. Immigrants had substantially higher rates of stomach and liver cancer mortality rates than the US-born, with the absolute risk of stomach and liver cancer mortality being particularly high among immigrant and US-born Asians, Hispanics, and blacks. Higher liver and stomach cancer mortality rates in these groups have been partly attributed to their higher incidence of hepatitis B virus and Helicobacter-pylori infection [7]. Detailed ethnic-nativity differentials in mortality from other major causes of death are reported elsewhere [7, 8].

The NVSS can be used to analyze all-cause and cause-specific mortality of immigrants in any age group. Besides data for broad racial/ethnic groups such as APIs, Hispanics, blacks, and whites, the NVSS allows analyses of immigrant mortality and life expectancy differentials for detailed Asian and Hispanic subgroups, such as Chinese, Japanese, Filipino, Asian Indians, Koreans, Vietnamese, Mexicans, Cubans, Puerto Ricans, and Central and South Americans [7, 8].

### 2.2. National Linked Birth and Infant Death File

National linked birth and infant death files are prepared by the NCHS as a byproduct of the natality and mortality components of the NVSS [23]. They are available as public-use data files for the 1983 through 2006 US birth cohorts and as period linked files from 2003 to 2009 [23–25]. In this dataset, the death certificate is linked with corresponding birth certificate for each infant who dies in the US. For each national birth cohort, approximately 30,000 infant deaths are linked to a cohort of more than 4 million births each year [23, 24].

The purpose of the linkage is to use many additional variables available from the birth certificate in infant mortality analysis [23]. Information on all 4.0 million births in the US each year is also included. For the 2002 birth cohort, more than 98% of US infant death certificates were successfully matched to their birth certificates. In the 2009 period-linked file, 982,942 live births and 4,644 infant deaths occurred among foreign-born mothers [23].

Besides nativity/immigrant status, the variables available for infant mortality and perinatal outcomes analyses include maternal age, race/ethnicity, marital status, education, place of residence, cause of death, age at death, birthweight, gestational age, tobacco and alcohol use during pregnancy, prenatal care utilization, maternal weight gain during pregnancy, and a variety of medical risk factors [23]. Nativity/immigrant status in the linked file is determined according to mother's place of birth as described in the natality file.

#### 2.2.1. Selected Results

Table 3 provides an analysis of nativity differentials in birth outcomes based on the linked file. Infants born to immigrant mothers have significantly lower risks of infant mortality, low birthweight, and preterm birth than those born to US-born mothers. Even after controlling for various infant- and maternal-risk factors, immigrants in most racial/ethnic groups experience lower infant mortality risks than natives. However, nativity patterns in birth outcomes and associated risk factors vary widely across racial/ethnic groups. In terms of absolute risk, several groups such as black immigrants and island/foreign-born Puerto Ricans have relatively high rates of infant mortality and low birthweight, while Asian Indian, Chinese, Filipino, black immigrants, and island/foreign-born Puerto Rican mothers are at higher risks of gestational diabetes (Table 3).

### 2.3. National Longitudinal Mortality Study (NLMS)

The National Longitudinal Mortality Study (NLMS) is a longitudinal dataset for examining socioeconomic, occupational, and demographic factors associated with all-cause and cause-specific mortality in the United States [18, 19, 26–30]. The NLMS is conducted by the National Heart, Lung, and Blood Institute in collaboration with the US Census Bureau, the National Cancer Institute, the National Institute on Aging, and the NCHS [26–29]. The NLMS consists of 30 Current Population Survey (CPS) and census cohorts between 1973 and 2002 whose survival (mortality) experiences were studied between 1979 and 2002 [28]. The CPS is a sample household and telephone interview survey of the civilian noninstitutionalized population in the United States and is conducted by the US Census Bureau to produce monthly national statistics on unemployment and the labor force. Data from death certificates on the fact of death and the cause of death are combined with the socioeconomic and demographic characteristics of the NLMS cohorts by means of the National Death Index [26–30]. Detailed descriptions of the NLMS have been provided elsewhere [26–30]. 

The NLMS consists of 2.7 million individuals drawn from 30 CPS and census cohorts whose mortality experience has been followed from 1979 to 2002. The total number of deaths during the 23-year followup is 341,343 [28]. Cancer incidence, stage of disease at diagnosis, and cancer survival data from 11 surveillance, epidemiology, and end results (SEER) cancer registries have also been linked to the various NLMS cohorts to prospectively study the risk of cancer incidence and mortality according to the baseline individual-level socioeconomic and demographic characteristics [31–33].

In the NLMS, place of birth (born in the 50 states, DC, US territories, Canada, Cuba, Mexico, or rest of the world) is the basis for defining nativity/immigrant status (US- or foreign-born) [28–30]. The NLMS does not include other immigration-related variables collected by CPS, such as citizenship/naturalization status and duration of residence in the US. For immigrant differentials in all-cause and cause-specific mortality, covariates such as age, race/ethnicity, marital status, rural/urban residence, education, occupation, employment status, family income, and housing tenure can be used [28–30]. The NLMS also permits analyses of early childhood social conditions as well as labor force transitions on risks of mortality from different causes of death. 

#### 2.3.1. Selected Results

**Figure 2:**
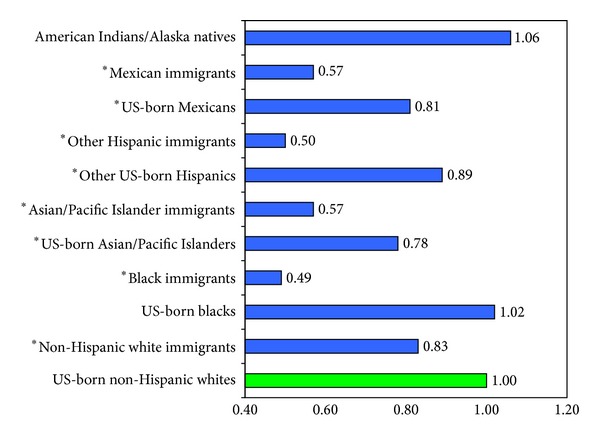
Ethnic-immigrant differentials in US all-cause mortality (hazard ratio or relative risk): The US National Longitudinal Mortality Study, 1980–1998 (*N* = 304, 594). Adjusted by Cox regression for age, sex, marital status, household size, education, family income, employment status, and rural/urban residence. *P < 0.05. US-born non-Hispanic whites were the reference group. Source: updated analysis of data presented [32].

**Figure 3:**
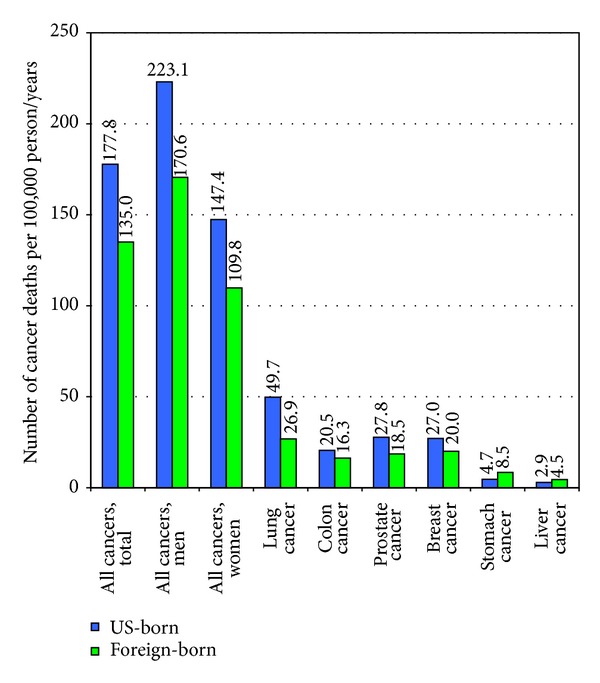
Site-specific US cancer mortality rates by nativity/immigrant status: The US National Longitudinal Mortality Study, 1980–1998 (*N* = 304, 594). Mortality rates are age-adjusted to the 2000 US standard population. Differences in mortality rates between US- and foreign-born individuals were statistically significant at the 0.05 level. Source: updated analysis of data presented in [31].

**Figure 4:**
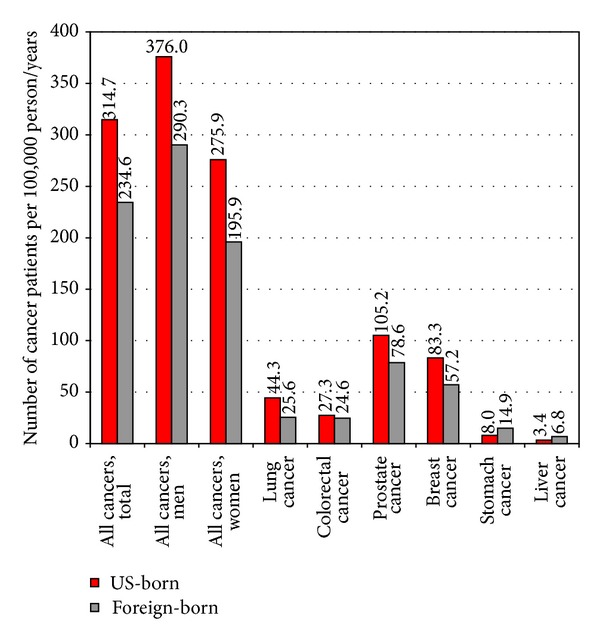
US cancer incidence rates by nativity/immigrant status: The US National Longitudinal Mortality Study Linked with 11 SEER Registries, 1980–1998. SEER: surveillance, epidemiology, and end results. Incidence rates are age-adjusted to the 2000 US standard population. Differences in incidence rates between US-born and foreign-born individuals were statistically significant at the 0.05 level for all cancers combined, lung, prostate, breast, and stomach cancers. The 11 SEER registries include Iowa, Hawaii, Seattle, Connecticut, Detroit, Utah, Los Angeles, San Francisco/Oakland/San Jose/Monterey, Greater California, Louisiana, and Kentucky.

According to the 1980–1998 NLMS, black, API, Mexican, and white immigrants aged ≥25 years had, respectively, 51%, 43%, 43%, and 17% lower risks of all-cause mortality than US-born non-Hispanic whites of equivalent socioeconomic and demographic background (Figure 2). Immigrants had significantly lower mortality rates than the native-born from all cancers combined and from lung, colorectal, prostate, and breast cancers. However, immigrants had substantially higher mortality rates than the native-born from stomach and liver cancers (Figure 3). The linked NLMS-SEER data indicate similar immigrant patterns in site-specific cancer incidence rates (Figure 4).

### 2.4. National Notifiable Diseases Surveillance System (NNDSS)

The NNDSS is a public health disease surveillance system administered by the CDC's Division of Notifiable Diseases and Healthcare Information [34]. All US states have laws requiring health providers, hospitals, and laboratories to report specific diseases to state and territorial jurisdictions for disease control and prevention purposes. The list of *reportable* diseases varies among states and over time. At the national level, the CDC identifies a list of *notifiable* diseases (http://wwwn.cdc.gov/nndss/script/downloads.aspx). *Notifiable* disease cases are reported on a voluntary basis by states to the NNDSS (without direct personal identifiers) for nationwide disease monitoring. NDSS also receives data directly from some CDC programs through separate notifiable disease reporting systems (e.g., tuberculosis, HIV, sexually transmitted diseases, and arboviral diseases) [34]. 

Researchers need to be aware of the characteristics and limitations of NNDSS data [35]. According to the CDC, disease reporting by states is likely incomplete, and completeness might vary by disease, time, and reporting state. Case definitions, surveillance approaches, and diagnostic capabilities may also vary by state and over time (http://wwwn.cdc.gov/nndss/script/casedefDefault.aspx). CDC publishes summarized notifiable diseases data from 57 local reporting jurisdictions weekly and annually in the Morbidity and Mortality Weekly Report (http://www.cdc.gov/mmwr/). Information on accessing more detailed NNDSS data can be found at http://isd-v-ncph-nnd/NNDSS/NNDSSLinkMain.html.

The number of immigration-related variables available from NNDSS varies by disease [36]. States may also add variables of interest to their routine data collection forms or during an outbreak or disease investigation. For many notifiable diseases (e.g., measles, pertussis, pneumococcal disease, *Haemophilus influenza*, polio, Lyme disease, cholera, listeriosis, and sexually transmitted diseases in adults), the CDC-developed reporting forms do not include any immigration-related information. For other diseases (e.g., dengue, viral hepatitis, and varicella), country of birth is collected. The HIV/AIDS Reporting System (HARS) by the CDC's Division of HIV/AIDS Prevention collects information on country of birth, country of residence at diagnosis, and birthplace of biological mother (for pediatric cases) [36, 37]. The CDC's National Tuberculosis Surveillance System collects more detailed immigration-related data within the NNDSS: country of birth, month-year of arrival in the US, country of birth for primary guardian(s), countries in which the patient has lived outside of the US for >2 months, under the custody of Immigration and Customs Enforcement at time of diagnosis, migrant/seasonal worker occupation, immigration status at first entry to the US, and moving out of the US to specific countries [38].

#### 2.4.1. Selected Results

**Figure 5:**
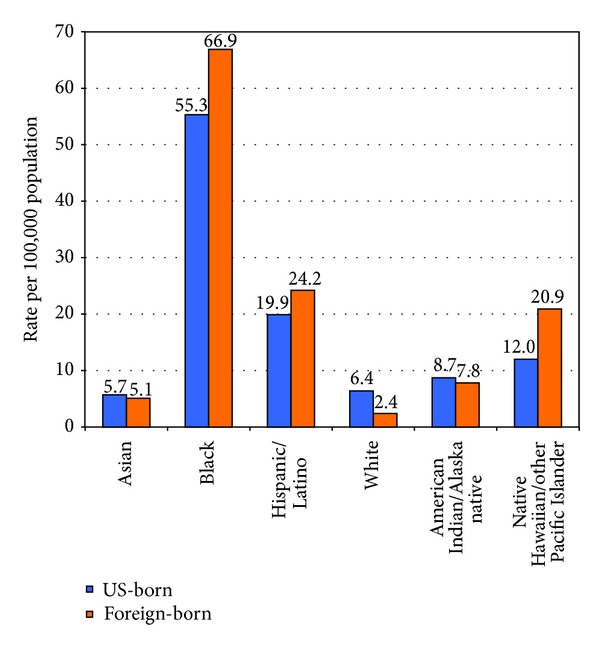
Rates of human immunodeficiency virus (HIV) diagnoses by race/ethnicity and nativity/immigrant status, 46 US states and 5 US territories, 2007–2010. Source: Prosser AT, Tang T, Hall HI. HIV in persons born outside the United States, 2007–2010, [17]. The relevant population denominator data are from the 2008–2010 American Community Survey. Estimated HIV numbers resulted from statistical adjustment that accounted for missing country of birth reporting delays and missing risk factor information, but not for incomplete reporting. For further details, see Prosser et al. [15]. Hispanics/Latinos can be of any race.

Approximately 16.2% of persons who received a diagnosis of HIV in the US and its territories during 2007–2010 were foreign-born, higher than the percentage-foreign-born (12.8%) in the general population [15]. Foreign-born blacks, hispanics, and native Hawaiians/other Pacific Islanders had higher HIV rates than their US-born counterparts, whereas the pattern was reversed for Asians, whites, and American Indians/Alaska natives (Figure 5).

The rate of new TB cases has been steadily decreasing in the US during the last two decades. In 2011, of 10,521 TB reported cases, 62.5% were foreign-born individuals. The TB rate of 17.3 per 100,000 population for foreign-born persons was 12 times greater than the rate for US-born persons (1.5 per 100,000). More than half of foreign-born persons with TB originated from five countries: Mexico, the Philippines, Vietnam, India, and China [39].

### 2.5. National Survey of Children's Health (NSCH)

The NSCH is conducted by NCHS, with funding and direction from the Maternal and Child Health Bureau [40–44]. The purpose of the survey is to provide national and state-specific prevalence estimates for a variety of children's health and well-being indicators [40–44]. The survey includes an extensive array of questions about the family, including parental health, stress and coping behaviors, family activities, and parental concerns about their children [40–44].

The 2011-2012 NSCH was a cross-sectional telephone survey conducted between February 2011 and June 2012 [41, 43]. The two previous rounds of the NSCH were conducted in 2003-2004 and 2007-2008 [42–45]. The 2011-2012 survey had a sample size of 95,677 children <18 years of age, including a sample of >1,800 children per state [41, 43]. In the NSCH, a random-digit-dial sample of households with children aged <18 is selected from each of the 50 states and DC. One child is selected from all children in each identified household to be the subject of the survey [40–45]. Interviews are conducted in English, Spanish, and four Asian languages. The respondent is the parent or guardian who knew most about the child's health status and health care. The interview completion rate for the 2011-2012 NSCH, a measure of the response rate indicating the percentage of completed interviews among known households with children, was 54.1% for the landline sample and 41.2% for the cell-phone sample [41, 43]. The interview completion rate was 66.0% in 2007 and 68.8% in 2003 [42, 44–49]. Substantive and methodological details of the NSCH are described elsewhere [42–49].

In NSCH, children's immigrant status can be defined by both children's own nativity and that of their parents [44, 46–48]. In the 2011-2012 NSCH, 15,826 children (26.4%) were born to immigrant parents. The NSCH includes primary language spoken in the home, and the 2007 survey contains data on child's and parents' length of stay in the US [40–42, 44].

#### 2.5.1. Selected Results

**Figure 6:**
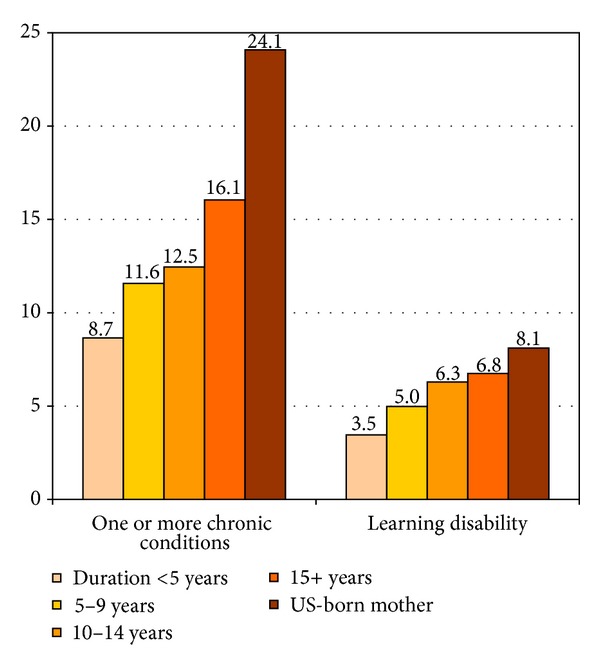
Prevalence (%) of chronic conditions and learning disability among children aged <18 years by mother's duration of residence in the United States, 2007. Source: The 2007 National Survey of Children's Health (*N* = 91, 642).

Table 4 shows nativity differentials in several behavioral and health outcomes among children and their parents. Immigrant children are defined here as those born to one or both immigrant parents. US-born children with both US-born parents are considered as the native-born. In 2011-2012, immigrant children aged 10–17 years were 24% more likely to be obese than native-born children. Immigrant children were substantially less likely than native-born children to engage in sports and physical activity. Immigrant children were less likely than native-born children to be diagnosed with behavioral problems, depression, autism, asthma, and attention deficit disorder/attention deficit hyperactivity disorder (ADD/ADHD). Interestingly, children's risk of having one or more chronic conditions and learning disability increased consistently in relation to mother's duration of residence in the USA (Figure 6). However, despite the lower prevalence of chronic conditions, immigrant parents were 2.2 times more likely than US-born parents to assess their children's general health as fair/poor. Immigrant parents were also more likely to report their own overall health as fair/poor compared to US-born parents (Table 4). 

**Figure 7:**
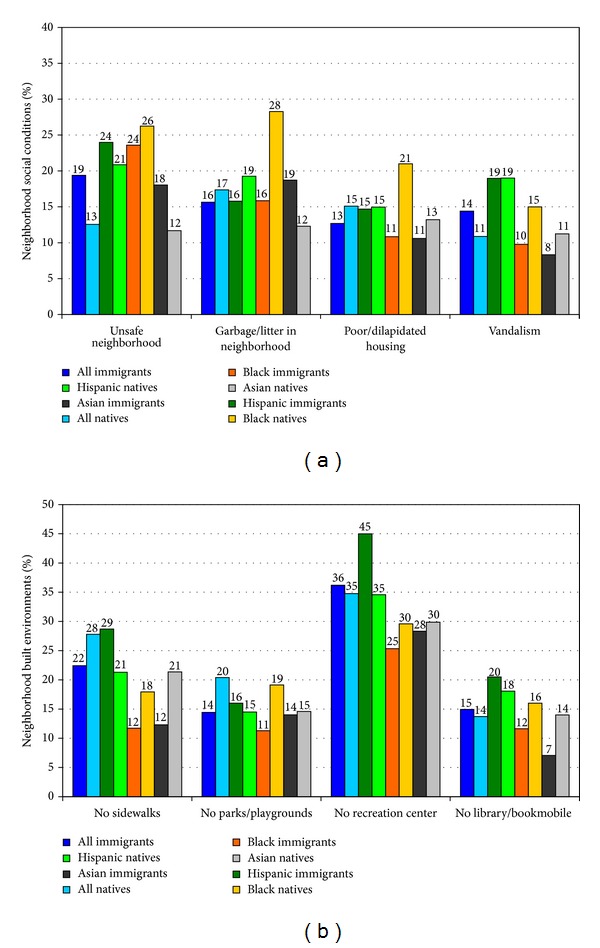
Neighborhood environments for immigrant and native-born children, United States, 2007. Source: The 2007 National Survey of Children's Health (*N* = 91, 642) Immigrant children: children of immigrant parents; native-born children: children of US-born parents.

Neighborhood conditions are often linked to inequalities in child and adult health. Immigrant children were more likely to live in unsafe neighborhoods or in neighborhoods characterized by vandalism such as broken windows and graffiti (Figure 7). However, nativity patterns in neighborhood social conditions varied by ethnicity. Neighborhood built environments also differed markedly for various ethnic-immigrant groups. Hispanic immigrant children were generally more likely than children in other groups to live in neighborhoods lacking sidewalks or walking paths, parks/playgrounds, recreation or community centers, libraries, or bookmobiles.

### 2.6. National Health Interview Survey (NHIS)

The NHIS is a national sample household survey in which data on socioeconomic, demographic, behavioral, morbidity, health, and healthcare characteristics are collected via personal household interviews [7, 50–52]. Data collected in the survey are based on self-reports. The survey uses a multistage probability design and is representative of the civilian non-institutionalized population of the United States. The NHIS is one of the longest running annual federal health surveys and is conducted by the NCHS [7, 12, 50]. Detailed descriptions of the NHIS can be found elsewhere [12, 50, 51]. The NHIS covers a broad range of health topics for both children and adults, including physical and mental health status, activity limitation, asthma, learning disability, ADHD, school absence, chronic conditions such as heart disease, cancer, diabetes, kidney disease, and liver disease, health-risk behaviors such as obesity, smoking, diet, physical inactivity, and alcohol use, health insurance coverage, and use of preventive health services such as cancer screening. Besides the core survey, the NHIS often includes supplemental surveys on special topics such as child health, mental health, cancer control, occupational health, child and adult immunization, complementary and alternative medicine, HIV, and diabetes [12, 50, 51]. 

In the NHIS, nativity/immigrant status is determined by place-of-birth information [7, 8, 52]. Besides immigrant status (US- or foreign-born), the public-use dataset includes geographic region of birth (USA; Mexico, Central America, Caribbean Islands; South America; Europe; Russia/former USSR; Africa; Middle East; Indian Subcontinent; Southeast Asia; and Asia), duration of residence in the US, and citizenship status (Table 1). In 2012, out of a sample of 108,131 children and adults, 18,560 were identified as immigrants.

#### 2.6.1. Selected Results

The NHIS is particularly useful for examining nativity/immigrant differentials in chronic-disease prevalence and risk factors [7, 52]. While immigrants were less likely to assess their general health as fair/poor than the US-born, the pattern varied greatly by ethnicity (Table 4). The risk of fair/poor health among adult immigrants increased with increasing length of stay in the USA. US-born blacks, Chinese immigrants, and Puerto Ricans were substantially more likely than US-born whites to assess their children's health as fair/poor. Among adults, island/foreign-born Puerto Ricans, Cuban immigrants, American Indians/Alaska Natives, and US-born blacks had the highest likelihood/prevalence of reporting their overall health as fair/poor (Table 5). 

Table 6 shows considerable variation in obesity and overweight prevalence among various ethnic-immigrant groups. Although immigrants in most racial/ethnic group had lower prevalence than their US-born counterparts, immigrants' risk of obesity and overweight increased with increasing duration of residence in the USA. In 2007–2012, obesity prevalence ranged from 3.1% for Chinese immigrants to 39% or higher for American Indians/Alaska natives, US-born blacks, native Hawaiians, and Pacific Islander immigrants. Approximately 70–80% of US-born blacks, US-born and foreign-born Mexicans, mainland US-born Puerto Ricans and island/foreign-born Puerto Ricans, American Indians/Alaska natives, native Hawaiians, and Pacific Islander immigrants were overweight or obese. After controlling for sociodemographic factors, compared to US-born whites, all Asian subgroups and black and white immigrants had significantly lower obesity risks, whereas US-born Mexicans, US-born blacks, American Indians/Alaska natives, Puerto Ricans, Native Hawaiians, and Pacific-Islander immigrants had significantly higher obesity risks.

Smoking rates vary widely among ethnic-nativity groups, with immigrants considerably less likely to smoke than the US-born (Table 6). Black immigrants were two-thirds less likely to smoke than US-born blacks (7.7% versus 21.7%), while Mexican immigrants were one-third less likely to smoke than US-born Mexicans (10.6% versus 16.5%). Immigrants' risk of smoking increased with increasing duration of residence in the USA. Even after controlling for various sociodemographic factors, ethnic-immigrant differentials remained with all Asian, Hispanic, and black immigrant groups reporting substantially lower smoking rates. Immigrants are more likely to be at a higher risk of physical inactivity than the US-born (Table 6). This pattern holds for all racial/ethnic groups except blacks. Rates of physical inactivity declined with increasing length of stay in the USA. Filipino, Asian Indian, and Cuban immigrants had 53%, 68%, and 86% higher adjusted odds of physical inactivity than US-born whites, respectively. 

### 2.7. National Health and Nutrition Examination Survey (NHANES)

During the past four decades, the NHANES surveys have been conducted periodically by the NCHS to obtain data on chronic disease prevalence and risk factors such as obesity, smoking, hypertension, cholesterol levels, diet and nutritional factors [12, 53]. Beginning in 1999, the NHANES became a continuous annual survey using a complex, stratified, and multistage probability clustered sample design, collecting data for a representative sample of the US civilian population. The NHANES data are based on clinical examinations, selected medical and laboratory tests, and in-home person interviews [12, 53]. 

The overall response rate in the NHANES for both interview and examination components was at least 76% in each of the six waves, 1999-2000, 2001-2002, 2003-2004, 2005-2006, 2007-2008, and 2009-2010. Substantive and methodological details of the NHANES are described elsewhere [12, 53].

Immigrant status in NHANES is derived by the country-of-birth variable (born in 50 US states or DC, Mexico, other Spanish-speaking country, or non-Spanish-speaking country). In the 2009-2010 NHANES, out of a total sample of 10,537 individuals, only 1,991 were foreign-born. The other immigration-related variables in the NHANES include naturalization/citizenship status and length of time in the USA (Table 1) [53]. 

#### 2.7.1. Selected Results

Because of small sample sizes, several years of NHANES data need to be pooled in order to conduct detailed ethnic and immigrant analyses, such as those in Table 7. Unlike NSCH and NHIS, obesity and overweight prevalence estimates for children, adolescents, and adults in NHANES are based on measured height and weight data. Table 7 shows lower obesity and overweight prevalence among foreign-born children aged 2–19 and adults aged ≥20 years compared to their US-born counterparts. Regardless of nativity, childhood and adult obesity prevalence among Mexicans and other Hispanics ranks among the highest in the world [52]. According to the 2001–2006 NHANES data, immigrants in each racial/ethnic group had lower total calorie and fat intake than the US-born. Moreover, immigrants' likelihood of excess calorie and fat intake increased with increasing length of residence in the USA [52].

### 2.8. American Community Survey (ACS)

Decennial censuses conducted by the US Census Bureau have long been the source of detailed socioeconomic and demographic information for the immigrant population in the United States [1, 4, 7, 8, 54]. With the discontinuation of the long-form questionnaire in the 2010 decennial census, the ACS has become the primary census database for producing socioeconomic, demographic, and housing characteristics of various population groups, including the immigrant population at the national, state, counties, and local levels [5, 54, 55]. The advantage of the ACS is that it is conducted annually with a sample size of over 3 million records, as compared with the decennial census long-form data, which were only available every 10 years [5, 55].

In the ACS Microdata Sample, nativity/immigrant status is derived from the place-of-birth variable, which provides extensive details on individuals' country of birth (Table 1) [2, 5]. Additionally, nativity of parents is available for children <18 years of age. The other immigration-related variables include duration of residence in the USA, naturalization/citizenship status, English language ability, and an extensive list of languages spoken at home [5]. By pooling multiple years of microdata samples, the ACS can be used to study socioeconomic, demographic, disability, and health insurance characteristics of various immigrant subgroups by cross-classifying the nativity status with the extensive race/ethnicity groupings that are available in the dataset. Summary statistics for select variables can also be obtained from the web-based American FactFinder [56].

#### 2.8.1. Selected Results

The 2011 Microdata Sample contains data on 349,161 immigrants, including information on 139,413 children born to immigrant parents. Numerous linguistic groups are represented in the 2011 Sample, including (unweighted frequency) data on 27,941 Chinese- (Mandarin and Cantonese) speaking and 5,166 Hindi-speaking individuals aged ≥5 years. Foreign-born children and working-age adults are, respectively, 24% and 52% less likely to have a disability (hearing, vision, cognitive, ambulatory, and self-care difficulties) than their US-born counterparts (Table 8). Child and adult disability rates are highest among those born in Puerto Rico and other US territories and lowest among those born in Asia and Africa. Immigrant children are 4.4 times more likely and working-age adults 2.1 times more likely than the US-born to lack health insurance. Approximately 41% of children, 52% of working-age adults, and 9% of elderly born in Latin America do not have health insurance coverage. More extensive nativity analyses of disability and health insurance are provided elsewhere [57].

## 3. Discussion and Directions for Future Research

In this paper, we have described eight major federal datasets and presented contemporary health statistics for various ethnic-immigrant groups in the United States. These data systems vary substantially in their coverage of health and behavioral characteristics, identification of ethnic and immigrant groups, time periods, data collection methodologies, and the types of data analyses that can be supported for studying immigrant health. Given the availability of a wide range of health variables and the inclusion of various ethnic-immigrant groups, the NVSS and NHIS are the two most important data systems for studying and monitoring immigrant health in the USA. These two data systems allow health, mortality, and morbidity estimates for some of the smallest and newest immigrant groups, reliable data for whom are not available elsewhere. The new and updated health, mortality, morbidity, and behavioral-risk data for immigrants presented herein should serve as the benchmark for setting up national health objectives for various immigrant groups in the USA and for conducting comparative analyses. 

Health, life expectancy, mortality, and morbidity patterns for immigrants and the native-born vary considerably in the USA. Overall, immigrants have better infant, child, and adult health, higher life expectancy, and lower disability and mortality rates than the US-born [7, 8, 24, 25, 29, 30, 44, 46, 48, 52, 57]. Nativity/immigrant patterns in several health outcomes, including those in mortality from major causes of death, vary across different racial/ethnic groups [7, 8]. Inequities in healthcare access and utilization between immigrants and the native-born are very marked [7, 8, 29, 57]. Acculturation, crudely measured by duration of residence since the time of immigration, plays a major role in modifying the social, behavioral, and health characteristics of immigrants, particularly of Asian and Hispanic immigrant groups, which generally leads to a decline in their health and mortality advantage over time [7, 8, 30, 46, 48, 52, 58].

A number of explanations have been suggested for higher life expectancy, better health, and lower mortality rates among immigrants. First, people immigrating to the USA may be healthier than those who remain in their countries of origin. This is referred to as the “healthy immigrant effect” or positive immigrant selectivity [7, 8, 29, 30, 44, 52]. Second, as shown here and elsewhere, immigrants have lower prevalence of health-risk behaviors than natives, including lower rates of smoking, drinking, obesity, and better diet [7, 8, 30, 44, 48, 52]. Third, immigrants appear to have higher levels of social and familial support and social integration compared to the native-born [7, 8, 24]. Fourth, socioeconomic characteristics might partly account for nativity differentials in health outcomes. Although immigrants are generally better educated, they have higher unemployment and poverty rates and lower rates of health insurance coverage than the US-born [7, 52, 57]. However, previous studies and analyses in this study indicate only a modest contribution of socioeconomic factors in explaining nativity differentials [7, 8, 24, 29, 30, 44, 52]. Lastly, inconsistencies in the coding of immigrant status in the numerator (mortality) and denominator (population) data may contribute to the reported life expectancy and mortality differentials between immigrants and the native-born [7, 8]. However, the NLMS and longitudinal cohort studies have produced mortality patterns consistent with the cross-sectional patterns based on the NVSS [7, 8, 29, 30].

Monitoring the health and well-being of immigrants is important not only in the United States but also in other industrialized countries with sizable immigrant populations such as Canada, Australia, the United Kingdom, Germany, France, Spain, Italy, and The Netherlands [7]. While the absolute number of immigrants in these countries is much smaller than that in the USA, the proportion of the foreign-born population is higher in Canada (20%), Australia (22%), and Spain (14%) than in the USA (13%) [59]. Several studies have documented immigrant health patterns in Canada and Europe [60–68].

Vital records and other administrative health databases in the USA generally do not contain several key immigration-related variables, such as duration of residence or recency of immigration, parental nativity status, citizenship/naturalization status, legal or refugee status, and English language proficiency, which may affect both immigrant health as well as its determinants [7, 8]. Population-based sample surveys can be a good source for facilitating in-depth analyses of these characteristics and other factors that influence immigrant health; however, they are not particularly useful for monitoring the health of many immigrant groups who represent a small proportion of the total population [7, 8]. Vital records, cancer registries, and other disease surveillance systems are important for identifying significant health problems and disease risks among various ethnic-immigrant groups, monitoring changes in their health status over time, and for etiological analyses [7, 8]. In the SEER cancer registries, more than 45% of all cancer patients' place-of-birth information is missing [33]. Analysis of nativity differentials in cancer incidence, disease stage, and survivorship based on cancer registries is biased because completeness of birthplace data in cancer registries varies systematically according to patient characteristics, including vital status [69, 70]. Clearly, such surveillance databases need to be strengthened and augmented with more complete reporting of birthplace data and additional information on the immigration process [7, 8]. Large national surveillance systems, such as the Behavioral Risk Factor Surveillance System and the Youth Risk Behavior Survey, do not include nativity or place-of-birth information for respondents; the inclusion of the nativity/immigration variable in these datasets would greatly improve the availability of and capacity to analyze a wide range of health, quality-of-life, and behavioral data on immigrants at the national, state, and local levels [71, 72]. Additionally, the data systems that link records from the major national population surveys with vital records and disease registries are particularly useful in this regard. Two national databases that use record linkages of population surveys with administrative sources, such as the National Death Index (NDI) and population-based cancer registries, are the ongoing NLMS and NHIS-NDI record linkage studies, which allow for complex analyses of immigrant health and mortality patterns [8, 28, 73–76]. With the continuation of long-term mortality followup, these longitudinal databases offer an exciting opportunity to analyze temporal changes in and determinants of immigrant health and mortality patterns.

## Figures and Tables

**Table 1:** National data systems for studying immigrant health in the United States.

Data system	Data collection and design	Government or sponsoring agency	Immigration variables	Period of data availability	Number of records or sample size	Subnational analysis	Advantages	Disadvantages
National Vital Statistics System (NVSS)	Period data; temporal; death and birth registration; complete count administrative data	National Center for Health Statistics, Centers for Disease Control and Prevention (CDC)	Decedent's nativity/immigrant status; maternal nativity status derived from place-of-birth variable	1900 through 2011	2.5 million deaths and 4.0 million births annually	Regions, census divisions, states, counties, and metropolitan and nonmetropolitan areas	Large number of vital records; race/ethnicity detail; geographic detail; long-term time trend; various health, mortality, and birth outcome measures	No data on several key immigration-related variables, for example, duration of US residence, naturalization, and English-language proficiency

National Linked Birth and Infant Death Files	Longitudinal; cohort; complete count administrative records	National Center for Health Statistics, Centers for Disease Control and Prevention (CDC)	Mother's nativity/immigrant status	1985 to 2009	30,000 to 40,000 infant deaths are linked to a cohort of more than 4 million births each year	Regions, census divisions, states, and counties	Large population size; ethnic detail; extensive infant mortality analysis by age, cause of death, and medical risks	No data on duration of US residence, naturalization, language, or legal status

National Longitudinal Mortality Study (NLMS)	Longitudinal; census and CPS records linked prospectively to deaths by cause of death and cancer incidence records	National Institutes of Health, US Census Bureau and National Center for Health Statistics, CDC	Nativity/Immigrant status; country/region of birth	1973 to 2008	2.7 million CPS records at baseline and 341,343 deaths during the 23-year mortality followup	State-level analysis possible for selected cohorts	Large sample size; self-reported race/ethnic detail; longitudinal; mortality by cause of death	Only a subset of the dataset is available as public-use file

National Notifiable Disease Surveillance System (NNDSS)	Surveillance conducted by health practitioners and laboratories at local, state, and national levels. State epidemiologists report cases of notifiable diseases to CDC, which tabulates and publishes these data on a weekly and annual basis	Office of Surveillance, Epidemiology, and Laboratory Services, Centers for Disease Control and Prevention (CDC)	It varies by specific disease or surveillance subsystem. For example, the Tuberculosis Surveillance System collects country of birth, year of arrival to the US, and country of birth for primary guardian(s), among others. For other notifiable diseases, no immigration variables are collected	1912 to present	It varies by disease and over time; for example, from 1 case of anthrax disease to 1.4 million *Chlamydia trachomatis *infections reported in 2011	Regions, states, and counties	National system; race/ethnicity detail; geographic detail; long-term time trend; various health outcome measures	Underreporting, state differences in surveillance approaches, changes in disease definitions, changes in the list of notifiable diseases over time and by state, and missing information

National Survey of Children's Health (NSCH)	Cross-sectional; sample survey; telephone survey	Health Resources and Services Administration (HRSA) and National Center for Health Statistics, CDC	Parents' and children's nativity/immigrant status; duration of residence in the US; English language proficiency	2003-2004, 2007-2008, and 2011-2012	Approximately 102,353 children under age 18 in 2003-2004, 91,642 in 2007-2008, and 95,677 children in 2011-2012	Regions, census divisions, and states	Large sample size; state-specific analyses; large number of health and behavioral indicators	All data based on parental reports; ethnic detail not available on the public-use file

National Health Interview Survey (NHIS)	Cross-sectional; temporal; sample survey; in-person interview data	National Center for Health Statistics, Centers for Disease Control and Prevention CDC	Children's and adults' nativity/immigrant status; duration of residence in the US; naturalization status; English language proficiency	1957 to 2012; immigrant status first became available in the 1976 survey	Approximately 100,000 children and adults annually	Four broad census regions only (northeast, midwest, south, and west)	Large sample size; race/ethnicity detail; long-term time trend; extensive sociodemographic, behavioral, health, and morbidity indicators	No geographic detail; data on most Asian subgroups suppressed on public-use file; no language variables; no information on immigrants' legal or refugee status

National Health and Nutrition Examination Survey (NHANES)	Cross-sectional; temporal; sample survey; in-person interview data	National Center for Health Statistics, Centers for Disease Control and Prevention CDC	Children's and adults' nativity/immigrant status; duration of residence in the US	1976 to 2010; periodic survey from 1976 to 1998; and continuous survey since 1999	Approximately 10,000 children and adults in each wave	None	Clinical examination data; medical and lab test results; measured height and weight	Small sample size; limited ethnic detail; no geographic detail; no language variables; no immigrants' legal or refugee status variable

American Community Survey, Public Use Microdata Sample (ACS)	Cross-sectional; sample survey; in-person interview	US Census Bureau	Nativity/immigrant status; parents' nativity status; detailed country-of-birth information; duration of residence in the US; naturalization status; English language ability; languages spoken at home	From 2000 to 2011	More than 3 million records in the annual sample	Regions, census divisions, states, and counties (on summary files)	Large sample size; extensive race/ethnicity detail; detailed country-of-birth information; language; naturalization status; duration of US residence	No health variables other than disability and health insurance coverage

**Table 2:** Average annual age-adjusted death rates for selected major causes of death by nativity/immigrant status, United States, 1999–2001.

Cause of death	Male					Female				
	US-born		Foreign-born		Rate	US-born		Foreign-born		Rate
	Rate	SE	Rate	SE	Ratio	Rate	SE	Rate	SE	Ratio
All-cause mortality	1092.80	0.60	846.60	1.59	0.77*	734.8	0.39	619.0	1.10	0.84*
Non-Hispanic white	1019.60	0.60	993.00	2.60	0.97*	717.3	0.42	716.3	1.79	1.00
Black	1463.80	2.31	883.30	8.17	0.60*	971.5	1.49	614.0	5.30	0.63*
Asian/Pacific Islander	744.80	6.13	666.60	3.51	0.89*	463.2	4.38	468.0	2.60	1.01
Hispanic	937.80	3.35	736.40	3.02	0.79*	604.4	2.25	507.2	2.00	0.84*
Cardiovascular diseases (CVD)	420.40	0.39	354.40	1.08	0.84*	286.3	0.24	274.6	0.70	0.96*
Non-Hispanic white	392.40	0.39	411.20	1.61	1.05*	278.0	0.26	304.0	1.00	1.09*
Black	533.90	1.48	353.20	5.45	0.66*	397.9	0.98	275.8	3.60	0.69*
Asian/Pacific Islander	310.70	4.09	279.60	2.39	0.90*	181.4	2.79	209.3	1.80	1.15*
Hispanic	339.20	2.18	293.00	2.04	0.86*	230.9	1.47	218.9	1.40	0.95*
All cancers combined	257.60	0.29	193.50	0.78	0.75*	169.3	0.20	135.8	0.53	0.80*
Non-Hispanic white	247.10	0.30	237.60	1.28	0.96*	169.6	0.22	171.4	0.94	1.01
Black	355.30	1.19	218.80	4.17	0.62*	203.7	0.70	140.4	2.42	0.69*
Asian/Pacific Islander	180.40	3.08	161.50	1.69	0.90*	122.2	2.33	107.2	1.14	0.88*
Hispanic	193.40	1.56	158.70	1.43	0.82*	119.4	0.99	104.6	0.91	0.88*
Stomach cancer	5.90	0.04	10.80	0.18	1.83*	2.90	0.03	5.80	0.11	2.00*
Non-Hispanic white	4.90	0.04	11.60	0.28	2.36*	2.40	0.03	5.40	0.16	2.27*
Black	13.00	0.23	14.20	1.05	1.10	6.40	0.13	7.30	0.57	1.15
Asian/Pacific Islander	13.90	0.86	12.50	0.47	0.90	7.30	0.56	7.50	0.31	1.03
Hispanic	10.60	0.37	8.90	0.33	0.83*	5.50	0.22	5.10	0.20	0.93
Liver and IBD cancer	6.40	0.05	9.80	0.17	1.52*	2.70	0.03	4.80	0.10	1.76*
Non-Hispanic white	5.80	0.05	7.40	0.24	1.29*	2.50	0.03	3.50	0.13	1.37*
Black	9.40	0.18	10.00	0.81	1.06	3.90	0.10	4.70	0.44	1.20
Asian/Pacific Islander	9.10	0.69	19.00	0.53	2.08*	4.10	0.42	8.10	0.31	1.98*
Hispanic	13.9	0.39	7.90	0.31	0.57*	4.80	0.20	5.20	0.21	1.07
Unintentional injuries	51.30	0.13	39.70	0.33	0.77*	22.90	0.07	16.90	0.21	0.74*
Non-Hispanic white	49.70	0.14	51.40	0.82	1.04*	23.20	0.09	23.00	0.54	0.99
Black	62.80	0.43	36.30	1.47	0.58*	23.60	0.23	15.30	0.89	0.65*
Asian/Pacific Islander	28.50	1.11	25.40	0.65	0.89*	13.10	0.70	14.10	0.44	1.08
Hispanic	48.80	0.61	42.50	0.55	0.87*	17.90	0.34	14.90	0.34	0.83*
Suicide	18.90	0.07	11.30	0.16	0.60*	4.20	0.03	2.90	0.08	0.69*
Non-Hispanic white	20.60	0.09	20.60	0.45	1.00	4.70	0.04	5.50	0.23	1.15*
Black	10.70	0.16	8.40	0.53	0.78*	1.80	0.06	1.50	0.20	0.84
Asian/Pacific Islander	13.20	0.72	8.70	0.31	0.65*	2.50	0.29	3.50	0.18	1.38*
Hispanic	11.90	0.28	9.20	0.27	0.78*	2.10	0.10	1.40	0.09	0.68*
Homicide	9.80	0.05	10.50	0.15	1.08*	3.00	0.03	2.60	0.10	0.86*
Non-Hispanic white	4.50	0.04	8.40	0.34	1.89*	2.10	0.03	2.90	0.21	1.36*
Black	39.50	0.29	22.90	0.84	0.58*	7.90	0.12	4.20	0.39	0.54*
Asian/Pacific Islander	4.60	0.38	5.70	0.24	1.24*	2.20	0.26	2.30	0.15	1.04
Hispanic	12.80	0.24	12.20	0.23	0.95	3.30	0.12	2.60	0.14	0.76*

Death rates are per 100,000 population and are age-adjusted by the direct method to the 2000 US standard population.

SE: standard error; Rate ratio: ratio of mortality rate for immigrants to that for the US-born. *P < 0.05. US- or native-born are individuals born in the 50 states, DC, Puerto Rico, and other US territories. Immigrants refer to those born elsewhere.

Source: [7].

**Table 3:** Infant mortality rates per 1,000 live births and prevalence (%) of sociodemographic and medical risk factors for selected ethnic-immigrant groups, United States, 1999–2002 (*N* = 16, 022, 367 live births).

Ethnic-nativity group	Teen birth (maternal age ≤ 19 years)	Maternal education ≥ 16 years	Delayed or no prenatal care	Smoking during pregnancy	Low birthweight	Preterm birth	Gestational diabetes	Chronic/pregnancy hypertension	Infant mortality rate	Unadjusted infant mortality risk ratio^4^	Adjusted infant mortality risk ratio^5^	95% confidence interval
Total population												
US-born^1^	12.47	26.22	3.23	14.27	8.01	12.16	2.86	5.09	7.21	1.39*	1.26	1.23–1.29
Foreign-born^2^	8.03	20.33	5.60	2.06	6.48	10.17	3.57	2.83	5.17	Reference	1.00	Reference
Non-Hispanic white												
US-born	8.81	32.35	2.19	16.02	6.78	10.75	2.88	5.16	5.74	1.25*	1.21	1.16–1.26
Foreign-born	3.25	41.33	3.48	5.67	6.00	9.32	3.06	3.89	4.59	Reference	1.00	Reference
Non-Hispanic black												
US-born	21.09	10.62	6.43	10.10	13.62	17.98	2.60	5.64	13.84	1.43*	1.41	1.36–1.48
Foreign-born	5.55	23.45	6.84	1.30	9.84	14.01	4.28	4.86	9.71	Reference	1.00	Reference
Chinese												
US-born	3.56	73.77	1.42	3.11	7.36	10.06	4.36	3.23	2.96	0.96	0.87	0.61–1.24
Foreign-born	0.58	54.12	2.24	0.32	5.06	7.24	4.91	1.33	3.08	Reference	1.00	Reference
Japanese												
US-born	3.40	58.23	1.45	5.65	8.02	10.80	5.34	4.14	5.37	1.47*	1.35	0.97–1.89
Foreign-born	0.43	48.29	2.28	2.91	7.08	7.43	2.39	1.41	3.65	Reference	1.00	Reference
Filipino												
US-born	12.57	32.92	3.05	6.89	9.41	12.89	3.84	4.03	5.84	1.04	0.94	0.78–1.15
Foreign-born	3.02	44.44	2.90	1.93	8.36	12.35	5.62	3.90	5.62	Reference	1.00	Reference
Asian Indian^3^												
US-born	6.17	60.69	3.61	4.04	10.03	11.02	3.97	2.93	7.28	1.81*	1.58	1.05–2.36
Foreign-born	1.09	54.70	3.89	0.26	9.45	9.87	8.00	2.44	4.02	Reference	1.00	Reference
Korean^3^												
US-born	8.59	56.04	2.87	8.05	6.38	9.63	3.00	3.38	3.26	0.83	0.70	0.34–1.46
Foreign-born	0.89	60.60	3.22	3.14	4.79	7.17	2.68	1.54	3.92	Reference	1.00	Reference
Vietnamese^3^												
US-born	27.30	17.66	6.19	10.70	7.72	10.39	2.24	2.74	4.80	1.22	1.26	0.60–2.63
Foreign-born	1.56	26.33	2.04	0.73	6.45	9.90	3.85	1.45	3.95	Reference	1.00	Reference
Mexican												
US-born	24.13	7.84	4.70	5.18	6.91	12.15	2.60	3.48	6.14	1.25*	1.30	1.25–1.35
Foreign-born	12.27	3.68	7.34	0.75	5.57	10.64	3.07	2.52	4.90	Reference	1.00	Reference
Puerto Rican												
Mainland US-born	21.32	9.32	4.65	11.15	9.47	13.77	3.54	3.73	8.33	1.05	1.00	0.91–1.10
Island/foreign-born	16.11	13.85	4.34	7.52	9.34	13.63	4.23	3.83	7.95	Reference	1.00	Reference
Central and South American												
US-born	20.31	21.81	3.52	4.78	7.34	11.25	2.40	3.67	5.52	1.15*	1.17	1.03–1.34
Foreign-born	8.05	13.54	5.51	0.94	6.34	11.19	3.06	3.06	4.82	Reference	1.00	Reference

^1^US-born are those born in the 50 states and the District of Columbia. ^2^Foreign-born are those born outside these territories. ^3^Data for Asian Indians, Koreans, and Vietnamese were available for only 11 states: CA, HI, IL, MI, MO, NJ, NY, TX, VA, WA, and WV. *P < 0.05. ^4^Risk ratio: ratio of the infant mortality rate or risk for the US-born in each ethnic group to that for the corresponding immigrant group. ^5^Adjusted by Cox proportional hazards regression for maternal age, marital status, birth order, infant sex, plurality, maternal education, prenatal care, and smoking during pregnancy.

Source: data derived from the 1999–2002 US National Linked Birth and Infant Death data files, see also [13].

**Table 4:** Weighted prevalence (%) and unadjusted odds ratios for selected behavioral and health indicators among children aged <18 years born to immigrant and US-born parents: The 2011-2012 National Survey of Children's Health (*N* = 95,677).

Behavioral or health indicator	Children of immigrant parents		Children of US-born parents		Odds for children of immigrant parents relative to children of US-born parents	
	%	SE	%	SE	OR	95% CI
Obesity (BMI ≥ 95th percentile)^a^	18.16	1.26	14.59	0.42	1.30	1.09–1.55
Overweight (BMI ≥ 85th percentile)^a^	34.39	1.48	29.89	0.54	1.23	1.07–1.41
No physical activity	14.36	0.87	7.08	0.26	2.20	1.88–2.58
Lack of sports participation	49.96	1.12	38.84	0.48	1.57	1.43–1.73
School absence > 2 weeks/year	2.83	0.32	7.30	0.28	0.37	0.29–0.47
Exposure to secondhand smoke	1.13	0.16	6.06	0.18	0.18	0.13–0.24
Fair or poor overall health status	5.17	0.42	2.35	0.12	2.27	1.85–2.77
Behavioral/emotional health problem	2.88	0.32	6.89	0.23	0.40	0.32–0.51
Depression	0.95	0.21	2.44	0.14	0.38	0.24–0.61
Autism spectrum disorder	1.29	0.22	2.18	0.13	0.59	0.41–0.85
Asthma	5.15	0.37	9.78	0.24	0.50	0.43–0.59
ADD/ADHD	2.91	0.29	9.42	0.26	0.29	0.23–0.36
Diabetes	0.27	0.08	0.48	0.06	0.56	0.29–1.05
Maternal breastfeeding rate	87.42	0.95	77.09	0.60	2.06	1.72–2.48
Mother in fair/poor health	16.22	0.67	10.34	0.26	1.68	1.50–1.88
Mother in fair/poor mental health	8.21	0.52	7.51	0.22	1.10	0.95–1.28
Father in fair/poor health	12.07	0.63	7.27	0.24	1.75	1.53–2.01
Father in fair/poor mental health	5.20	0.42	4.42	0.18	1.19	0.98–1.43
Parental/household smoker	14.22	0.61	27.30	0.36	0.44	0.40–0.49

ADD/ADHD: attention deficit disorder/attention deficit hyperactivity disorder.

Nativity differences in prevalence were statistically significant at *P* < 0.01 for all indicators except diabetes and mother's and father's mental health status.

^a^Defined for children and adolescents aged 10–17 years.

**Table 5:** Weighted prevalence and adjusted odds of parent- or self-assessed fair or poor health among US children and adults from 26 ethnic-immigrant groups: The National Health Interview Survey, 2006–2012.

Ethnic-immigrant group	Children under 18 years (*N* = 164,105)				Adults aged 18+ years (*N* = 447,024)			
	Prevalence		Adjusted odds ratio^1^		Prevalence		Adjusted odds ratio^1^	
	%	SE	OR	95% CI	%	SE	OR	95% CI
Duration of residence in the US (years)								
<5	1.87	0.28	0.72	0.53–0.97	6.21	0.35	0.51	0.45–0.57
5–9	2.27	0.31	0.82	0.62–1.09	6.88	0.30	0.53	0.48–0.58
10–14	2.24	0.35	0.79	0.57–1.10	9.07	0.35	0.66	0.60–0.72
15+	2.28	0.71	0.86	0.46–1.60	15.45	0.25	0.87	0.83–0.91
US-born	1.87	0.05	1.00	Reference	12.69	0.13	1.00	Reference
Ethnic-immigrant group								
Non-Hispanic white, US-born	1.20	0.06	1.00	Reference	11.81	0.15	1.00	Reference
Non-Hispanic white, immigrant	0.88	0.29	0.66	0.34–1.27	11.08	0.42	0.88	0.81–0.95
Non-Hispanic black, US-born	3.39	0.13	1.89	1.67–2.14	18.60	0.27	1.48	1.41–1.54
Non-Hispanic black, immigrant	1.42	0.46	0.73	0.37–1.44	9.28	0.41	0.72	0.65–0.79
American Indian/Alaska native	2.67	0.77	1.56	0.88–2.78	20.38	1.00	1.65	1.41–1.93
Asian Indian, US-born	0.89	0.25	0.92	0.52–1.62	2.39	0.88	0.58	0.26–1.28
Asian Indian, immigrant	0.74	0.39	0.54	0.19–1.57	6.07	0.46	0.65	0.56–0.75
Chinese, US-born	0.82	0.26	0.76	0.41–1.42	4.82	1.01	0.69	0.43–1.10
Chinese, immigrant	2.46	1.20	2.05	0.75–5.65	8.60	0.65	0.62	0.54–0.72
Filipino, US-born	1.02	0.31	0.96	0.52–1.75	9.08	0.94	1.00	0.80–1.24
Filipino, immigrant	0.29	0.22	0.22	0.05–0.96	9.28	0.57	0.79	0.69–0.92
Hawaiian/Pacific Islander, US-born	2.99	1.74	2.07	0.66–6.45	8.91	1.83	0.91	0.63–1.29
Pacific Islander, immigrant	2.98	2.11	1.60	0.37–6.92	11.57	3.37	0.83	0.48–1.45
Other Asians, US-born^2^	1.67	0.25	1.26	0.93–1.71	8.00	1.28	0.94	0.73–1.21
Other Asians, immigrant^2^	1.19	0.37	0.73	0.39–1.35	11.40	0.48	0.89	0.81–0.98
Mexican, US-born	2.69	0.13	1.54	1.32–1.81	12.82	0.32	1.36	1.28–1.44
Mexican, immigrant	3.19	0.32	1.29	1.01–1.64	13.71	0.35	0.98	0.91–1.04
Puerto Rican, mainland US-born	3.52	0.34	2.04	1.61–2.60	12.62	0.66	1.61	1.42–1.81
Puerto Rican, Puerto Rico-born	6.33	1.67	2.87	1.60–5.15	24.08	0.92	1.58	1.42–1.76
Cuban, US-born	2.17	0.66	1.60	0.87–2.94	6.33	0.77	1.01	0.79–1.29
Cuban, immigrant	4.58	2.34	1.90	0.67–5.37	20.93	1.15	1.04	0.92–1.17
Central and South American, US-born	1.91	0.21	1.14	0.89–1.48	5.42	0.50	0.97	0.80–1.18
Central and South American, immigrant	1.85	0.46	0.92	0.55–1.54	12.46	0.37	0.90	0.83–0.97
Other Hispanics, US-born	3.14	0.47	2.12	1.54–2.91	13.99	0.75	1.29	1.13–1.48
Other Hispanics, immigrant	0.49	0.50	0.25	0.03–2.01	13.18	1.94	0.97	0.71–1.31
All other groups	1.78	0.37	1.43	0.93–2.20	9.23	0.69	0.83	0.70–0.98

OR: odds ratio; SE: standard error; CI: confidence interval.

^1^Adjusted by logistic regression model for survey year, age, gender, ethnic-immigrant status (or race/ethnicity and length of immigration), region of residence, and poverty status.

^2^This category includes Koreans, Vietnamese, Japanese, Cambodians, Laotians, Hmongs, Thais, Pakistanis, and other Asians.

**Table 6:** Weighted prevalence and adjusted odds of health-risk behaviors among US adults aged 18+ years from 26 ethnic-immigrant groups: The National Health Interview Survey, 2007–2012.

	Current smoking				Physical inactivity				Obesity (BMI ≥ 30)				Overweight (BMI ≥ 25)			
Ethnic-immigrant group	Prevalence		Adjusted odds ratio^1^		Prevalence		Adjusted odds ratio^1^		Prevalence		Adjusted odds ratio^1^		Prevalence		Adjusted odds ratio^1^	
	%	SE	OR	95% CI	%	SE	OR	95% CI	%	SE	OR	95% CI	%	SE	OR	95% CI
Duration of residence in the US (years)																
<5	11.8	0.7	0.58	0.50–0.67	42.0	1.2	1.34	1.21–1.50	11.8	0.8	0.38	0.33–0.45	44.7	1.3	0.57	0.51–0.64
5–9	10.6	0.6	0.47	0.41–0.54	43.9	1.0	1.31	1.19–1.45	16.7	0.7	0.46	0.41–0.51	50.9	1.0	0.56	0.51–0.61
10–14	9.9	0.6	0.43	0.37–0.49	41.9	0.9	1.17	1.07–1.28	19.9	0.8	0.53	0.48–0.59	58.1	1.0	0.70	0.64–0.76
15+	11.9	0.3	0.65	0.60–0.70	40.6	0.5	1.03	0.98–1.09	24.3	0.4	0.68	0.64–0.73	63.6	0.5	0.84	0.79–0.88
US-born	21.2	0.2	1.00	Reference	32.3	0.4	1.00	Reference	28.9	0.2	1.00	Reference	63.3	0.2	1.00	Reference
Ethnic-immigrant group																
Non-Hispanic white, US-born	21.5	0.2	1.00	Reference	30.6	0.4	1.00	Reference	26.8	0.2	1.00	Reference	61.5	0.2	1.00	Reference
Non-Hispanic white, immigrant	15.7	0.7	0.80	0.73–0.89	31.3	0.8	1.11	1.02–1.21	20.0	0.7	0.73	0.67–0.80	56.1	0.9	0.84	0.78–0.90
Non-Hispanic black, US-born	21.7	0.4	0.65	0.61–0.68	42.6	0.6	1.33	1.26–1.41	39.3	0.5	1.66	1.59–1.73	72.0	0.4	1.81	1.73–1.90
Non-Hispanic black, immigrant	7.7	0.7	0.21	0.17–0.25	41.6	1.4	1.45	1.29–1.63	22.0	1.1	0.75	0.66–0.84	62.6	1.3	1.12	1.01–1.26
American Indian/Alaska native	32.0	2.7	1.20	0.91–1.57	38.4	2.2	1.14	0.92–1.40	41.7	2.1	1.87	1.57–2.23	69.6	1.8	1.54	1.29–1.85
Asian Indian, US-born	4.4	1.5	0.26	0.13–0.52	20.8	4.0	1.23	0.79–1.94	8.6	2.7	0.42	0.21–0.83	39.3	5.2	0.81	0.52–1.27
Asian Indian, immigrant	5.6	0.6	0.31	0.24–0.39	31.2	1.6	1.68	1.43–1.98	10.4	1.1	0.38	0.30–0.48	46.0	1.6	0.60	0.52–0.69
Chinese, US-born	4.7	1.2	0.29	0.18–0.48	22.6	2.8	1.25	0.89–1.74	7.0	1.4	0.30	0.20–0.45	32.6	2.7	0.43	0.34–0.55
Chinese, immigrant	6.0	0.6	0.28	0.22–0.36	34.2	1.5	1.40	1.20–1.63	3.1	0.6	0.09	0.06–0.14	24.3	1.3	0.21	0.18–0.24
Filipino, US-born	17.4	1.9	0.86	0.65–1.13	30.4	2.2	1.25	1.01–1.53	20.9	1.9	0.81	0.64–1.02	55.8	2.6	0.93	0.75–1.15
Filipino, immigrant	10.6	1.1	0.57	0.45–0.73	33.3	1.9	1.53	1.29–1.81	11.9	1.1	0.41	0.33–0.51	49.9	1.9	0.68	0.59–0.79
Hawaiian/Pacific Islander, US-born	23.9	3.8	0.94	0.62–1.43	30.3	4.8	1.30	0.81–2.10	40.4	3.9	2.13	1.53–2.96	72.9	4.3	2.40	1.51–3.82
Pacific Islander, immigrant	13.0	4.7	0.37	0.17–0.81	37.6	6.7	1.27	0.66–2.45	41.3	6.5	1.81	1.02–3.20	79.6	5.0	2.40	1.18–4.90
Other Asians, US-born^2^	15.3	1.3	0.80	0.66–0.98	24.2	1.6	1.08	0.92–1.27	13.0	1.3	0.54	0.42–0.68	44.9	1.7	0.68	0.58–0.79
Other Asians, immigrant^2^	13.4	0.9	0.54	0.46–0.64	38.6	1.2	1.46	1.30–1.64	6.2	0.6	0.18	0.15–0.22	32.0	1.2	0.29	0.26–0.33
Mexican, US-born	16.5	0.6	0.48	0.44–0.52	33.5	0.9	1.14	1.06–1.24	37.3	0.8	1.75	1.64–1.87	71.7	0.6	2.01	1.88–2.15
Mexican, immigrant	10.6	0.4	0.19	0.17–0.20	48.3	0.8	1.28	1.19–1.37	29.4	0.6	0.96	0.90–1.03	71.7	0.7	1.54	1.43–1.66
Puerto Rican, mainland US-born	20.1	1.3	0.57	0.48–0.67	40.7	1.8	1.48	1.27–1.71	35.5	1.8	1.57	1.35–1.82	70.0	1.6	1.82	1.57–2.12
Puerto Rican, Puerto Rico-born	17.2	1.3	0.50	0.42–0.60	54.4	1.6	1.58	1.36–1.84	34.7	1.3	1.30	1.15–1.46	71.2	1.4	1.52	1.33–1.73
Cuban, US-born	19.2	2.6	0.78	0.54–1.10	32.0	3.2	1.40	1.03–1.90	25.9	3.9	1.10	0.73–1.65	59.3	2.9	1.23	0.95–1.60
Cuban, immigrant	15.4	1.3	0.51	0.42–0.63	56.9	1.7	1.86	1.59–2.19	28.2	1.6	0.92	0.79–1.07	67.6	2.0	1.15	0.94–1.41
Central & South American, US-born	11.5	1.5	0.38	0.28–0.51	29.9	2.2	1.29	1.04–1.60	24.1	2.0	1.16	0.93–1.46	60.1	2.2	1.56	1.28–1.91
Central & South American, immigrant	9.4	0.6	0.20	0.18–0.24	47.8	1.0	1.42	1.29–1.55	22.0	0.8	0.68	0.62–0.75	64.1	0.9	1.10	1.01–1.19
Other Hispanics, US-born	23.6	1.5	0.91	0.76–1.08	28.2	1.5	0.91	0.76–1.08	33.7	1.9	1.46	1.23–1.73	73.0	1.5	1.97	1.68–2.31
Other Hispanics, immigrant	12.3	2.6	0.34	0.21–0.55	39.8	4.1	1.22	0.86–1.73	34.4	4.2	1.32	0.91–1.92	71.0	3.4	1.41	1.04–1.92
All other groups	17.9	2.1	0.69	0.52–0.92	31.7	2.5	1.17	0.92–1.49	26.5	2.6	1.16	0.87–1.55	58.6	2.7	1.11	0.88–1.40

OR: odds ratio; SE: standard error; CI: confidence interval.

^1^Adjusted by logistic regression for survey year, age, gender, ethnic-immigrant status (or race/ethnicity and length of immigration), region of residence, education, marital status, poverty status, and occupation.

^2^This category includes Koreans, Vietnamese, Japanese, Cambodians, Laotians, Hmongs, Thais, Pakistanis, and other Asians.

**Table 7:** Obesity and overweight prevalence (weighted) among US children and adolescents aged 2–19 years (*N* = 16, 717) and adults aged 20+ years (*N* = 18, 391) by immigrant status: The 1999–2006 National Health and Nutrition Examination Survey (NHANES).

Nativity/immigrant status	Childhood obesity prevalence		Childhood overweight prevalence		Adult obesity prevalence		Adult overweight prevalence	
	%	SE	%	SE	%	SE	%	SE
Total population	**15.4**	**0.5**	**31.3**	**0.9**	**31.8**	**0.7**	**65.7**	**0.6**
US-born	15.7	0.5	31.6	0.8	33.4	0.7	66.6	0.7
Foreign-born	12.2	1.1	24.9	1.5	22.9	1.0	60.9	1.1
Non-Hispanic white								
US-born	13.5	0.8	29.5	1.2	31.1	0.7	64.8	0.8
Foreign-born	10.0	2.5	16.8	3.6	24.2	2.3	57.8	2.5
Non-Hispanic black								
US-born	19.4	0.7	35.2	0.8	44.2	1.0	74.0	0.9
Foreign-born	13.8	2.4	24.3	2.8	21.9	2.5	61.4	2.1
Mexican American								
US-born	21.9	1.0	38.3	1.2	40.2	1.7	73.5	2.0
Foreign-born	16.8	1.2	35.6	1.5	28.7	1.4	70.7	1.2
Other Hispanic								
US-born	20.4	2.1	37.8	2.3	38.3	4.4	72.3	3.7
Foreign-born	12.2	3.2	26.9	4.5	26.8	1.9	70.8	2.6
All other ethnic groups								
US-born	13.4	1.6	26.9	2.5	42.1	4.1	73.3	3.0
Foreign-born	4.9	2.2	12.8	3.7	6.5	1.7	35.2	2.6

Childhood overweight and obesity are defined as body mass index (BMI) at or above the gender- and age-specific 85th and 95th percentile cut-off points from the 2000 CDC growth charts, respectively, and age-specific 85th and 95th percentile cutoff points from the 2000 CDC growth charts, respectively. Adult overweight is defined as body mass index (BMI) ≥25 and obesity as BMI ≥30. Obesity and overweight prevalence in NHANES are based on measured height and weight data.

**Table 8:** Rates (weighted%) of disability and no health insurance coverage among US children, working-age adults, and elderly according to nativity/immigrant status and world region of birth: The 2011 American Community Survey, Public Use Microdata Sample (*N* = 3, 112, 017).

	Disability			No health insurance coverage		
	<18 years	18–64	≥65	<18 years	18–64	≥65
Nativity/immigrant status						
Foreign-born	3.1	5.5	36.3	29.2	38.4	5.5
US-born	4.1	11.4	38.9	6.7	18.0	3.1
World region of birth						
US-born (50 states and DC)	4.1	11.4	38.9	6.7	18.0	3.0
Puerto Rico and US Island territories	10.8	17.2	45.8	8.6	21.5	1.1
Latin America	3.3	6.1	39.3	40.7	52.2	8.8
Asia	2.3	4.5	34.5	12.7	21.1	4.8
Europe	4.3	6.9	35.7	7.7	17.5	1.8
Africa	2.3	4.9	31.8	14.2	28.5	9.3
Northern America (Canada and Mexico)	3.0	6.7	31.6	8.0	12.0	1.3
Oceania	4.2	5.1	31.0	17.8	22.0	2.4
